# An Effective Method to Prepare Curcumin-Loaded Soy Protein Isolate Nanoparticles Co-Stabilized by Carrageenan and Fucoidan

**DOI:** 10.3390/ph17040534

**Published:** 2024-04-21

**Authors:** Yaxin Chen, Shuyun Cai, Niaoniao He, Xiaomei Huang, Zhuan Hong, Jianlin He, Hui Chen, Yiping Zhang

**Affiliations:** 1 School of Chemical Engineering, Huaqiao University, Xiamen 361021, China; chenyaxin462@163.com; 2 Technical Innovation Center for Utilization of Marine Biological Resources, Third Institute of Oceanography, Ministry of Natural Resources, Xiamen 361005, China; sycai7@163.com (S.C.); 13887434960@163.com (N.H.); hzh@tio.org.cn (Z.H.);; 3 College of Pharmacy, Fujian University of Traditional Chinese Medicine, Fuzhou 350122, China; 4 School of Marine Biology, Xiamen Ocean Vocational College, Xiamen 361005, China; hhuangxiaomei@xmoc.eud.cn

**Keywords:** curcumin, soy protein isolate, composite nanoparticle, sulfate-based anionic polysaccharide, 3D double-layer network

## Abstract

In this study, a novel and simple strategy is proposed based on 3D network formed by easily blending polysaccharide carrageenan (Car) and fucoidan (Fuc) without a crosslinker. The Fuc/Car dual coating effectively assists the self-assembly of soy protein-isolated (SPI)/curcumin (Cur, C) composite microcapsules (SPI/C) and achieves an excellent curcumin encapsulation efficiency (EE) up to 95.28% with a 4.16% loading capacity (LC) under optimal conditions. The resulting nanocomposites achieved a satisfying redispersibility in aqueous solution and enhanced the water solubility with a lower size dispersity index (PDI) of 0.12 and a larger zeta potential of −29.67 mV. The Fuc/Car double-layer network not only dramatically improved its thermal stability and photostability, but also provided controlled release and enhanced antioxidant activity in in vitro conditions. The underlying mechanism of the self-assembly of the curcumin-loaded nanoparticles was also addressed. The results proved the feasibility of the encapsulation of unstable hydrophobic bioactive substances (curcumin) with the dual anionic polysaccharide Fuc/Car co-stabilized SPI nanoparticles. This study paves the way for an alternative way of developing novel curcumin delivery systems and will have broad prospects in the pharmaceutical industries.

## 1. Introduction

Curcumin (Cur, C) is the main phenolic compound in the rhizome of turmeric. The chemical structure of curcumin is “bis-feruloylmethane”, where α,β-diketone serves as the main chain with symmetrical o-methylphenol attached at both ends [1]. Increasing evidence indicates that curcumin has a wide range of pharmacological activities [2], such as anti-inflammatory, antioxidant, and free radical scavenging activities [3]. Hence, it possesses immense potential to be developed into a therapeutic drug.

However, its oral availability is low due to its high hydrophobicity and poor stability, which is susceptible to light and heat, limiting its application in the pharmaceutical industry.

To overcome these drawbacks, nano-/micro-delivery systems are used for enhancing the entrapment, stability, and controlled release of curcumin. Current research studies on curcumin delivery systems include liposome [4], microemulsion [5], micelles [6], and nanoparticles [7]. In particular, nanoparticles have drawn tremendous attention for their unique advantages [8], such as small size, good water solubility, and ability to improve the water dispersibility of hydrophobic compounds. Protein carriers such as bovine serum albumin, ovalbumin [9], casein [1], and soy protein isolate (SPI) [10,11] are used in the preparation of curcumin nanoparticles.

In particular, SPI, a plant protein, is widely available, inexpensive, and hypoallergenic compared with animal protein [10]. Unfortunately, the high positive charge density of SPI means that it easily agglomerates under neutral or even low pH aqueous solutions due to the reduced electrostatic repulsion [12].

The electrostatic interactions between polysaccharides and proteins could be used to stabilize protein-based drug carriers [13,14]. Differently to previous research which utilized a combination of cationic polymers and anionic polysaccharides to simply construct layer via electrostatic interaction, which is unstable and discontinuous, we constructed a new type of curcumin-loaded SPI nanoparticle that is co-stabilized by a carrageenan (Car)/fucoidan (Fuc) double coating. It was surprising to find that non-gelling polysaccharide Fuc could interact with Car to form a 3D network structure using rich polar groups without a crosslinker, such as sulfated groups, hydroxyl, and carboxyl which are able to enhance the hydrophilicity and stability of curcumin-loaded SPI.

As a natural anionic polysaccharide, Car can neutralize the positive charge on the surface of SPI at a low pH to produce stable Car composite nanocarriers [15]. Fuc, a fucosyl-sulfated polysaccharide, can modulate the overall charge of the composite nanoparticles because of its higher charge density, and its addition could result in the nanoparticles having a broader pH adaptability and a higher ionic strength tolerance [16], which is conducive to further improving the redispersibility of the composite nanoparticle. In addition, the use of Fuc to synergistically enhance the function of carried active substances like curcumin’s antioxidant, hypoglycemic, and anti-inflammatory activities is promising [17].

Therefore, the purpose of this study was to better stabilize curcumin-loaded SPI nanoparticles and achieved an improved controlled release and enhanced antioxidant properties via a dual-anionic polysaccharide coating. This innovative approach to stabilizing curcumin-loaded SPI nanoparticles with a Fuc/Car-reinforced 3D coating network without a crosslinker would be an effective method with good prospects for functional food or drug applications. The results that prove our concept are presented in the following section.

## 2. Results and Discussion

### 2.1. Effects of the SPI-to-Car Mass Ratio on the Properties of the Nanoparticles

The average particle size and zeta potential of a composite nanoparticle are two crucial indicators that can be used to measure the stability of a system. The smaller the average particle size, the better the dispersion of particles in the aqueous solution. Zeta potential can be used to predict the stability of the dispersive system. Generally speaking, a larger absolute value of the zeta potential indicates that there are stronger repulsive forces between particles and that the system is more stable.

As shown in Table 1, the nanoparticle sizes were measured by dynamic light scattering according to different SPI-to-Car ratios (*w*/*w*). The particle size of the SPI/Car nanoparticles was 1069.49 nm without adding Car. However, when Car was added, the particle size decreased significantly (*p* < 0.05) to an SPI-to-Car ratio of 1:0.25 (*w*/*w*), which was around 344.04 nm. This phenomenon was consistent with Su’s results, which indicated that the negatively charged Car could be deposited on the surface of a positively charged SPI to avoid the mutual integration of SPIs due to hydrophobic interaction. The average particle size gradually decreased when the mass ratio of the SPI-to-Car ratio was in the range of 1:0.25 to 1:1, and the minimum particle size was obtained when the mass of the SPI was equal to Car. This may be because the positive charge on the SPI can be completely saturated by anionic polysaccharide Car, and the excess negative charge on the surface of SPI can effectively avoid aggregation due to the electrostatic repulsion [18]. The results of zeta potential also supported this phenomenon. After adding Car, the zeta potential changed from a positive potential (17.58 mV) to a negative potential (−18.17 mV), increased gradually to the ratio of 1:0.25–1:1, and then remained almost constant. Perhaps, the addition of Car destroyed the hydrophobic effect of SPI, thus unfolding more SPI structures with exposed amino groups, and the zeta potential was increased due to the growing repulsive forces between SPI particles.

Moreover, the higher the turbidity, the higher the yield of nanoparticles formed by complex coacervation in the system [19]. The turbidity first increased and then decreased with a mass ratio that increased from 1:0 to 1:1.5 (Table 1), and the turbidity at the mass of 1:1 remained at its maximum, which is similar to the results for the zeta potential. Therefore, we chose 1:1 as the best SPI-to-Car mass ratio.

### 2.2. Effect of pH on the Properties of the Nanoparticles

The amount of opposite charge between proteins (SPI) and polysaccharides (Car) is the key to their complexation, which is determined by the pH of the system. The pH chosen in our experiment was between the pKa of SPI (4.5) and the pKa of Car (2.5) so that these two polymers could interact electrostatically with the opposite charges [20].

As shown in Table 2, with decreasing pH, the particle size of composite nanoparticles decreased initially and then increased. The zeta potential increased significantly (*p* < 0.05) from a pH of 4.5 to a pH of 3.5, and then it decreased to be within the range of pH 3 to pH 2.5, which indicates that the system reached relative stability at the pH of 3.5. Meanwhile, the turbidity of the system decreased significantly (*p* < 0.05) when the pH was too low (2.5) or high (4.5) and no significant difference (*p* > 0.05) in turbidity was observed at pH 3.0, 3.5, or 4.0. This is because the closer the pH of the system was to the isoelectric point of each polymer, the lower the degree of ionization of SPI and Car, leading to poor dispersibility.

The effect of pH on the morphology of the Car/SPI nanoparticles is presented in Figure S2. The nanoparticles gradually formed in the solution as the pH decreased. The near-circular outline of the nanoparticles was clearly observed at the pH of 3.5. When the solution was at a lower pH of 3.0, many flocculent materials (Car and SPI) appeared and began to coalesce excessively, resulting in precipitation at the pH of 2.5. These results corroborated our analysis of the effects of physicochemical properties on particle size and zeta potential; that is, when the pH was 3.5, the particle size and zeta potential reached the desired minimum value and maximum value, respectively. Hence, the ideal pH for complex coacervation between SPI and Car was 3.5.

### 2.3. Effects of the SPI-to-Curcumin Mass Ratio on the Properties of the Nanoparticles

As shown in Table 3, the particle size of curcumin-loaded Car/SPI nanoparticles showed a significant increase compared to those without curcumin (Table 1). This may be because curcumin is encapsulated by the nanoparticles, which have an influence on the average size of the particles. At 50:1 to 5:1 of SPI-to-curcumin mass ratios, the particle size gradually decreased from 420.41 nm to 417.88 nm, which showed a statistically significant difference (*p* < 0.05). Then, it slightly increased to a mass ratio of 3:1. The variation in the encapsulated efficiency in the range of 50:1 to 5:1 was identical to the particle size with an increasing addition of curcumin, indicating that the particle size is correlated with the encapsulation of curcumin, which again verified the above speculation.

At mass ratios of 5:1 and 3:1, the encapsulated efficiency was considerably decreased (*p* < 0.05), indicating that a relatively large amount of curcumin was unencapsulated by the Car/SPI carrier. Meanwhile, adding curcumin caused an increase in the negative zeta potential of nanoparticles in the ratio range of 50:1 to 10:1. The turbidity of the aqueous dispersions increased accompanied by an increase in the curcumin, which confirmed that the excess of curcumin could hardly be loaded by the nanoparticles and only accumulated in the aqueous solution.

Combining the results of Table 3, the mass ratios of 25:1 and 10:1 were considered, but they were not statistically different (*p* > 0.05) from each other considering their physicochemical properties. Taking the yield of nanoparticles and the increase in curcumin into account, we finally chose 10:1 as the optimal ratio of SPI to curcumin.

### 2.4. Effects of the SPI-to-Fuc Mass Ratio on the Properties of the Nanoparticles

Fuc is a sulfated anionic polysaccharide with a strong electronegativity due to the abundance of carboxyl, sulfate, and other polar groups in its structure, which ensure that it has a good dispersibility in aqueous solutions under neutral conditions. Therefore, it could be deposited onto the surface of the composite cohesive product, the Car/SPI/C nanoparticles, through hydrogen bonding or electrostatic interactions, forming a more stable particle structure.

Table 4 shows that the addition of Fuc significantly increased (*p* < 0.05) the zeta potential, turbidity, and loading capacity of nanoparticles, indicating that the Fuc could noticeably improve the physicochemical properties of the nanoparticles. Figure S3 shows that the particle size and encapsulation efficiency of the nanoparticles were increased compared to nanoparticles without Fuc. This may be because the wider pH range and higher ionic strength tolerance of Fuc can further be deposited onto the surface of Car/SPI/C to facilitate the stability of composite nanoparticles via electrostatic attraction and hydrogen bonding in a self-assembly manner, and similar work also found this phenomenon [21]. On the other hand, the compact structure and low penetration characteristics of Fuc make it possible for it to form a 3D-like gel structure connected with Car. According to the previous work described by Sriprablom et al. [22], the shear modulus of emulsions with the increased addition of Gum Arabic showed an increasing trend. The G′ (storage modulus) was much larger than the G′′ (loss modulus), implying that all the emulsion samples were dominated by elastic behavior and mainly formed an elastic-like network structure, which could exhibit a higher mechanical strength.

In addition, from the inserted photo in Figure S3 it can be seen that the nanoparticle without Fuc was more clearly precipitated compared to the nanoparticles with different mass ratios of Fuc (from 1:0.1 to 1:0.5), indicating that Fuc inhibited the aggregation of complex coacervating nanoparticles (Car/SPI/C) by providing more negative charges [19,23], leading to a higher dispersion and turbidity. However, the reason for the decreased load capacity was presumably the increased weight caused by Fuc coating, resulting in a smaller proportion of curcumin in the overall nanoparticle.

Considering that there were no significant differences (*p* > 0.05) in turbidity or loading capacity between the mass ratios of 1:0.4 and 1:0.5 and that the encapsulation efficiency reached the maximum at a mass ratio of 1:0.4 (Figure S3), we finally chose 1:0.4 as the optimal mass ratio of SPI to Fuc.

### 2.5. Particle Size, Zeta Potential, PDI, and Redispersibility of the Nanoparticles

In our study, the SPI/C, Car/SPI/C, and Fuc/Car/SPI/C nanoparticles were fabricated at optimal conditions (5 mg/mL of curcumin, 1:1 SPI/Car mass ratio, pH 3.5, 10:1 SPI/curcumin mass ratio, 1:0.4 SPI/Fuc mass ratio) and compared with neat curcumin prepared under the same conditions (as a control group). The final encapsulation efficiency and loading capacity of the optimized nanoparticles (Fuc/Car/SPI/C) were 95.28% and 4.16%, respectively (data shown in Figure S3 and Table 2).

**Figure 1:**
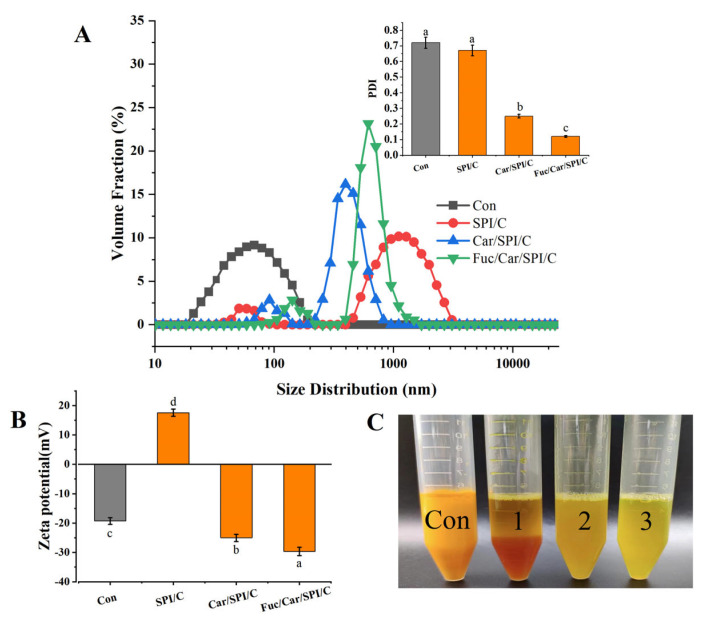
The particle size, PDI (**A**), zeta potential (**B**), and a photo of the redispersed lyophilized samples in water (**C**). Con: pure curcumin; 1: SPI/C nanoparticles; 2: Car/SPI/C nanoparticles; 3: Fuc/Car/SPI/C nanoparticles. Different letters in panel B mean significant difference between groups.

The particle size distributions of the different samples are presented in Figure 1A. There was a notable right shift between the different samples in terms of the particle size distribution. The average particle sizes of each sample were ordered as follows: SPI/C > Fuc/Car/SPI/C > Car/SPI/C > C. Since SPI/C particles tend to aggregate, the particle size measured was the largest, greater than 1 µm, which is consistent with the results in Table 1. The particle size distributions of the Car/SPI/C and Fuc/Car/SPI complex nanoparticles were much narrower than those of the SPI/C and control samples, which indicated that the particle size in the composite nanocapsules was more uniform. Our results agree with those obtained by Peng et al. [24]. Furthermore, the PDI results presented in the inserted figure show a decreased PDI, particularly a significant decline between the Car- or Fuc-coated nanoparticles and SPI/C. This result was corroborated by the zeta potential measurement shown in Figure 1B. It was found that the zeta potential increased considerably both on the Car/SPI/C and Fuc/Car/SPI compared to the positively charged SPI/C (*p* < 0.05), and there were significant differences between Car/SPI/C and Fuc/Car/SPI/C. This finding indicates that the negatively charged Fuc was thoroughly deposited on the surface of Car/SPI/C to further stabilize the curcumin-loaded nanoparticles. Meanwhile, in Figure 1C, sample 1 (SPI/C) and the control sample (curcumin) were investigated as they formed clear precipitates at the bottom of the centrifugal tubes. By contrast, there were no precipitates in sample 2 (Car/SPI/C) or sample 3 (Fuc/Car/SPI/C), and the latter dispersed more uniformly and stably in water, verifying that using a Car/Fuc double coating was conducive to further improving the redispersibility of curcumin-loaded SPI nanoparticles in water. According to the previous study [21], this phenomenon can be attributed to the novel construction of Fuc/Car/SPI/C, which we will further confirm with the SEM images.

### 2.6. Morphology Analysis of the Nanoparticles

**Figure 2:**
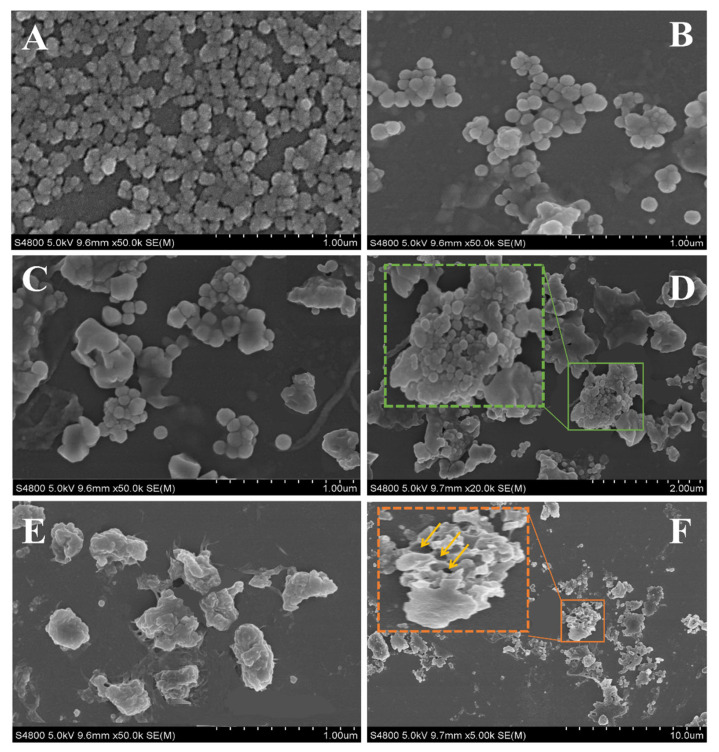
SEM images of nanoparticles. (**A**): SPI nanoparticles; (**B**): SPI nanoparticles loaded curcumin; (**C**,**D**): Car/SPI/C nanoparticles at different magnifications; (**E**,**F**): Fuc/Car/SPI/C nanoparticles at different magnifications. The green box and orange show the uncoated region and cross-section of nanoparticles, respectively; The yellow arrow points to core holes in nanoparticles.

SEM images of SPI, SPI/C, Car/SPI/C, and Fuc/Car/SPI/C are shown in Figure 2. As depicted in Figure 2A, numerous SPI nanoparticles clustered together forming aggregates, which highlighted the viewpoint that we mentioned previously: that the positive charge carried by SPI facilitates aggregation through hydrophobic interactions. The problem of the instability of the SPI was clearly not alleviated using an antisolvent precipitation with the addition of a curcumin–ethanol solution homogenized at high speed (Figure 2B) [10], which ties in well with the results for the PDI and zeta potential in “Section 2.5”.

To improve the stability of SPI/C, Car and Fuc/Car were added to form the composite nanoparticles shown in Figure 2C–F. Although the Car membrane structure was gradually formed to conceal some SPI/C nanoparticles through complex coacervation (Figure 2C), a number of unsealed SPI/C nanoparticles still existed (Figure 2D). This is probably because the reaction process of complex coacervation was reversible without crosslinkers, leading to a discontinued membrane being formed by Car and SPI after the solution was left standing for a while. After coating with Fuc, the surfaces of the nanoparticles became more irregular and rougher. To better understand the interior structure of Fuc/Car/SPI/C, a cross-section of nanoparticles was investigated from the broken nanoparticle in Figure 2F, and many “core holes” (yellow arrow points to) were observed. This finding indicated that the self-assembled nanoparticles were composed of multinucleated composite microcapsules due to numerous SPI/C nanoparticles being encapsulated by the Car/Fuc co-stabilized layer, which was consistent with our previous speculation that Fuc and Car mainly formed a novel 3D dual-layer network construction, and the numerous sulfate, carboxyl, and hydroxyl groups on Car and Fuc offered adequate connection points for the SPI/C nanoparticles. The mechanism was elaborated in the similar work showing that two polysaccharides microphase-separate into internal buffer region (hard SSPS) and external restricted region (soft HA) via supramolecular interactions, resulting in a double-layer network (SHA), which highlighted the strong connection and dispersion capability of an SHA binder [25]. Other scholars confirmed that anionic polysaccharides can break the interactions between super absorbent polymer and water molecules [26,27], thus leading to self-assembly between Fuc and Car through hydrogen bonding, ion–dipole, and electrostatic interactions instead.

**Figure 3:**
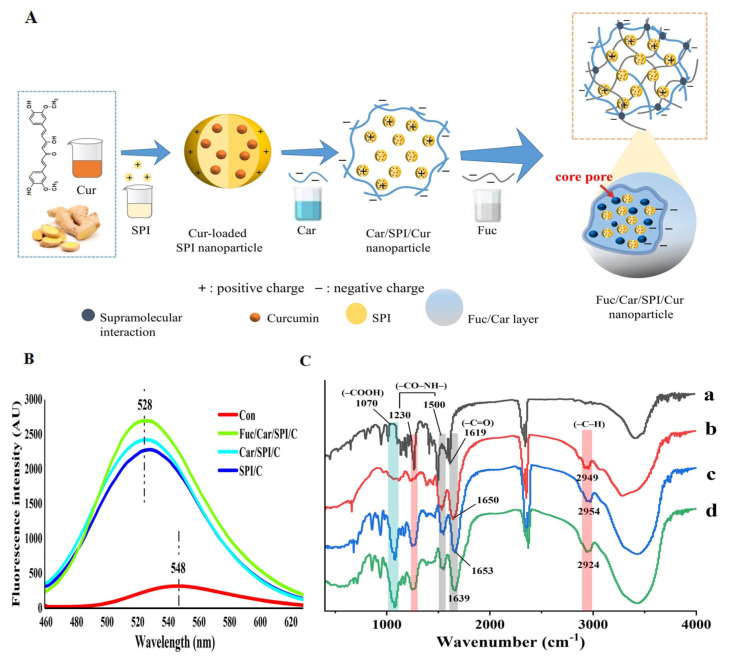
Morphological analysis of the nanoparticles. (**A**) A schematic diagram of the self-assembly mechanism of the curcumin-loaded nanoparticle; (**B**) fluorescence spectra of curcumin in different samples. (**C**) (a): curcumin; (b): SPI/C; (c): the physical mixture of the Fuc, Car, SP, I and C is present in the nanoparticles in equivalent ratios; (d) Fuc/Car/SPI/C.

According to the SEM results, we created a schematic that reflects how the nanoparticles are self-assembled. The details are shown in Figure 3A. The overall positively charged curcumin-loaded SPI nanoparticles, formed by the hydrophobic interaction between curcumin and SPI, were not stable. Thus, a simple strategy was proposed based on the dual anionic polysaccharides (Car/Fuc) coated with self-assembled 3D networks.

Firstly, the negatively charged groups on the carrageenan, such as carboxyl group, could easily form electrostatic interactions with the positive amino group on the surface of the curcumin-loaded SPI through a pH adjustment (the optimized pH was 3.5), accordingly blocking the contact sites on SPI particles. Next, the incorporated Fuc could easily combine with Car in the aqueous solution as it introduced functional groups (e.g., sulfate groups, carboxyl group) to their side chains; therefore, the polymer chains within the 3D network layer could be formed via supramolecular interactions (e.g., ion–dipole, hydrogen bonding, electrostatic interactions, etc.) [25]. This also offers adequate connection points within themselves and with curcumin-loaded SPI particles.

To validate the mechanism behind the association between the curcumin and the composite nanoparticles (SPI/C, Car/SPI/C, Fuc/Car/SPI/C), the fluorescence emission spectra of curcumin was used and the results are shown in Figure 3B; as can be seen, the free curcumin diluted with distilled water had a relatively broad fluorescence emission peak at approximately 548 nm, with a considerably low fluorescence intensity. This may be because the free curcumin was surrounded by an extremely polar aqueous environment, leading to the unstable fluorescence of the curcumin, which was easily subjected to extinction by the solution’s components. After the curcumin was encapsulated in the SPI nanoparticles, its fluorescence intensity was dramatically increased, and there was a blue shift in the position of the emission peak from 548 nm to 528 nm. This finding indicated that the encapsulation of curcumin by SPI nanoparticles can change the association environment of curcumin from hydrophilic to hydrophobic [28], which greatly improved the stability of the curcumin fluorescent. In particular, the fluorescence emission intensity of the curcumin was further enhanced when it was encapsulated in the dual anionic polysaccharides network structure (Fuc/Car), and the fluorescence emission peaks were further shifted toward shorter wavelengths compared to those of Car/SPI/C and SPI/C. Similar results were also reported by Liu et al. [26]. This phenomenon demonstrates that the novel Fuc/Car coating can provide a more hydrophobic conjugation environment for SPI/C or curcumin, which could effectively validate the mechanistic diagram shown in Figure 3A.

The characteristic peaks of curcumin include the C=O stretching vibration at 1619 cm^−1^ and the amide bond in the region of 1400–1600 cm^−1^. The characteristic peak of SPI is located at 1230 cm^−1^, which can be attributed to the unique amide band of proteins and the stretching vibrations of the C-H hydrophobic groups at 2949 cm^−1^. Polysaccharides exhibit characteristic peaks at 1070 cm^−1^ due to the symmetric stretching vibrations of the carboxyl group (vibrations of -COOH). It is clearly observable that the characteristic peaks of curcumin are present in all three nanoparticle samples (indicated by the black areas). However, in comparison to SPI/C, the nanoparticles coated with the Fuc/Car complex exhibited a blue shift in the C-H hydrophobic group vibration (from 2949 to 2924 cm^−1^) and in the C=O stretching vibration (from 1650 to 1639 cm^−1^), which is indicative of a significant reduction in the hydrophobicity of the nanoparticles after Fuc/Car coating. However, these changes are not observed in the physical mixtures of the three components, and the intensity of the carboxyl vibration peaks of the nanoparticles coated with disaccharides is relatively diminished. These alterations confirm that the proteins and polysaccharides are not simply physically mixed but are conjugated through electrostatic interactions between the amino groups of the proteins and the carboxyl groups of the polysaccharides.

### 2.7. Superior Stability of the Fuc/Car/SPI/C

**Figure 4:**
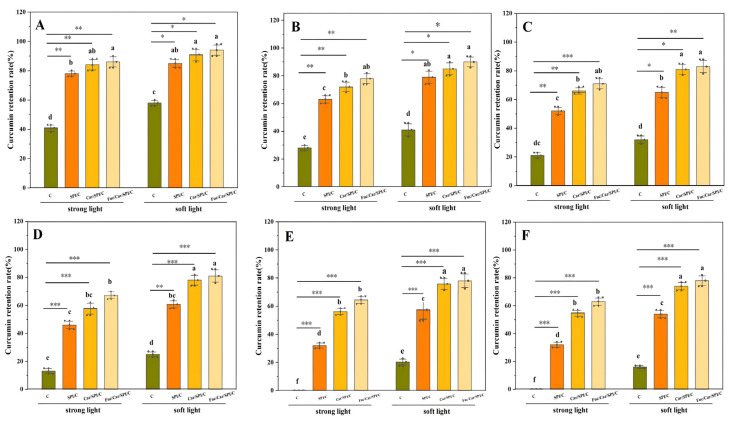
The photochemical stability of the samples. (**A**): after 8 h of strong light and 16 h of soft light; (**B**): after 16 h of strong light and 32 h of soft light; (**C**): after 24 h of strong light and 48 h of soft light; (**D**): after 32 h of strong light and 64 h of soft light (**E**): after 40 h of strong light and 80 h of soft light (**F**): after 48 h of strong light and 96 h of soft light. * indicates a significant difference between groups: * *p* < 0.05, ** *p* < 0.01, *** *p* < 0.001. C represents pure curcumin.

Firstly, the photochemical stability of the nanoparticles was determined as the curcumin retention is a crucial factor. The results for the samples (C, SPI/C, Car/SPI/C, Fuc/Car/SPI/C) that were exposed to strong light and soft light for different periods of time are shown in Figure 4.

As expected, the retention of curcumin encapsulated in nanoparticles was significantly higher (*p* < 0.05, *p* < 0.01) than that of unencapsulated curcumin (in Figure 4A–C). Meanwhile, most of the curcumin in the control group was degraded (in Figure 4D–F) after the irritation with a strong light (48 h) or after an extended period (96 h) of irradiation with a soft light. In addition, the retention of curcumin in Fuc/Car/SPI/C was slightly increased compared with that of Car/SPI/C nanoparticles, probably because the addition of Fuc increased the scattering of light and reduced the loss of curcumin to light exposure.

**Figure 5:**
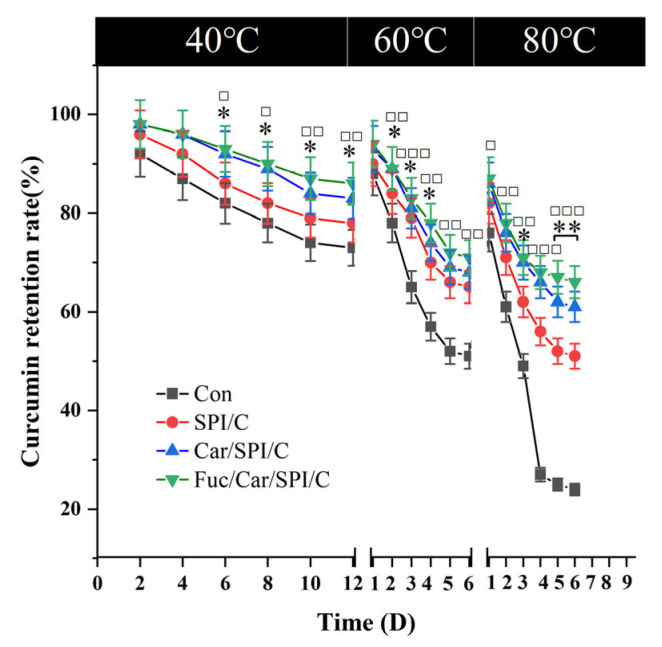
The thermal stability of the samples at 40 °C, 60 °C, and 80 °C, respectively. * represents a significant difference between groups of Fuc/Car/SPI/C and SPI/C: * *p* < 0.05, ** *p* < 0.01; □ means a significant difference between groups of Fuc/Car/SPI/C and Con: ^□^ *p* < 0.05, ^□□^ *p* < 0.01,^□□□^ *p* < 0.001. Con represents pure curcumin.

Secondly, the thermal stability of the nanoparticles was assayed (shown in Figure 5). The retention of curcumin was determined at different temperatures (40 °C, 60 °C, and 80 °C) and compared with the control group (free curcumin). We also accidently found that the curcumin retention between Car/SPI/C and Fuc/Car/SPI/C nanoparticles was not considerably different. This may be because the SPI/polysaccharide composite nanoparticles provide a similar hydrophobic environment for curcumin in contrast to the transition from encapsulated to unencapsulated curcumin. Other researchers found similar phenomena using a Tremella polysaccharide with zein [29] or zein mixed with PGA/rhamnolipid [30]. Future studies should further investigate the self-assembled curcumin-loaded SPI nanoparticle co-stabilized with Fuc/Car.

### 2.8. Better Controlled Release and Antioxidant Activity of Fuc/Car/SPI/C

**Figure 6:**
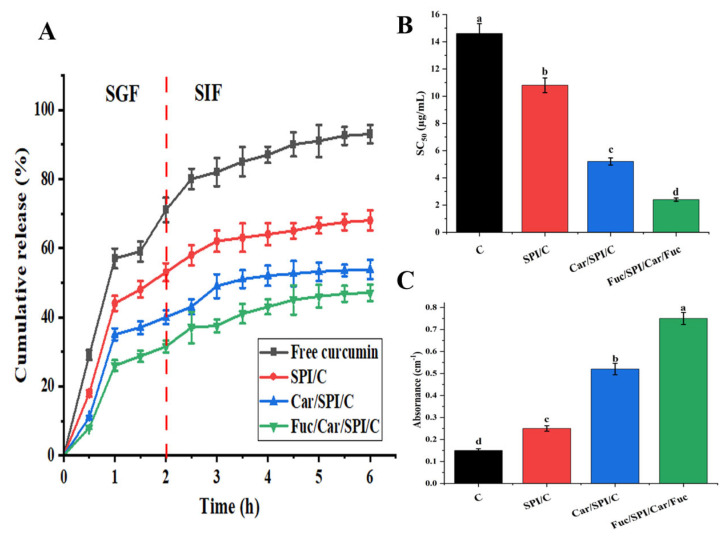
The kinetic release (**A**), DPPH radical scavenging activity (**B**), and reducing power (**C**) of different samples including curcumin dispersed in distilled water. The content of curcumin is consistent with composite nanoparticles. The different letters mean significantly different (*p* < 0.05).

The kinetic release profiles of curcumin in SPI/C and the composite nanoparticles (Car/SPI/C, Fuc/Car/SPI/C) were studied consecutively under simulated gastric and intestinal conditions, as shown in Figure 6A. During the entire SGF digestion period, 71% of the free curcumin was released; despite the curcumin being controlled to some extent in SPI/C, 53% of the curcumin still diffused into the SGF. In contrast, 40% and 31% of the curcumin in the composite nanoparticles was released, which were 13% and 22% less than in the former conditions. Upon entering the SIF digestion period, free curcumin was continuously released, whereas the release rate in the nanoparticles was significantly reduced. Among them, Fuc/Car/SPI/C had the best controlled release effect and only around 47% of the curcumin was released into the SGF.

To better understand the mechanism of curcumin release in the Fuc/Car/SPI delivery system, the release profiles of hydrogels were successfully fitted onto Korsmeyer–Peppas Kinetics with a high correlation coefficient (R^2^ = 0.99). This result was consistent with the report by Gupta et al. [31]. The result of the model indicated that the k and n values were 22.73 and 0.44. The n values were lower than 0.45, so the release mechanism was clearly Fickian diffusion.

A DPPH radical scavenging test and reducing power assay were employed to determine the in vitro antioxidant activity of different samples, as shown in Figure 6B,C. The results revealed a higher antioxidant activity for Fuc/Car/SPI/C compared to pure curcumin and other nanoparticles, which is reflected in the significant decrease in SC_50_ (*p* < 0.05) and remarkable increase (*p* < 0.05) in the reducing power. This can be justified by the enhanced supramolecular interactions (including ion–dipole, hydrophobic interaction, and electrostatic interactions) between Fuc and Car-stabilized SPI nanoparticles due to sulfates and the other polar groups provided by Fuc. In accordance, Wang et al. [32] also reported that Fuc and Car form a strong entanglement network compared to non-gelling Fuc, and this is speculated to be the main reason for the improvement in antioxidant activity.

Despite the advantages of the curcumin-loaded SPI nanoparticles co-stabilized by carrageenan and fucoidan, there are still some drawbacks: 1. It is necessary to further study the effects of the 3D network on the other biological activities of curcumin. 2. The formation of a 3D network structure is only preliminarily expounded, and the supramolecular interactions’ mechanism is unclear. An attractive approach is using more detailed characterization methods (such as rheology, FTIR, Cryo-SEM, etc.) to study the ability of this transport carrier and expand it to other hydrophobic drugs or bioactive components. These will be our future research objectives.

## 3. Materials and Methods

### 3.1. Materials

SPI (protein content ≥ 97%) and curcumin (purity ≥ 70%) were purchased from Sigma-Aldrich Reagents Co., Ltd. (St.Louis, MO, USA). Car (κ-type, CAS number: 11114-20-8) was purchased from Shishi Globe Agar Industry Co., Ltd. (Quanzhou, China), and sulfate content was detected to be 21.6%. Fuc was prepared in our laboratory (40.1% purity). All other chemicals were of analytical grade. 1, 1-Diphenyl-2-picrylhydrazyl (DPPH) free radicals were purchased from Tokyo Chemical Industry (Tokyo, Japan).

### 3.2. Preparation of the Nanoparticles

#### 3.2.1. Car-Stabilized SPI Nanoparticles under Different Car/SPI Ratios

The nanoparticles were prepared in accordance with a previously reported method [18] with slight modifications. The 5 mL of distilled water containing SPI (10 mg/mL) was poured into 5 mL of Car solution (0, 2.5, 5, 10, 12.5, 15 mg/mL), which was also made with distilled water. The pH of the system was adjusted to 3.5 with 0.1% (*v*/*v*) acetic acid solution while stirring, followed by homogenization with a high-speed shear homogenizer (10,000 rpm) for 10 min. The final aqueous dispersion of Car/SPI composite nanoparticles was obtained, where the final concentration of SPI was 5 mg/mL and the mass ratios of SPI to Car were maintained at 1:0, 1:0.25, 1:0.5, 1:1, 1:1.25, and 1:1.5.

#### 3.2.2. Car-Stabilized SPI Nanoparticles under Different pH

We poured 5 mL of SPI solution (10 mg/mL) into 5 mL of Car solution (10 mg/mL). The pH was adjusted to 2.5, 3, 3.5, 4, and 4.5 while stirring. Then, the solution was homogenized for 10 min using a high-speed shear homogenizer (10,000 rpm). The final concentration of SPI was 5 mg/mL.

#### 3.2.3. Curcumin-Loaded Car-Stabilized SPI Nanoparticles (Car/SPI/C) under Different Curcumin Content

Curcumin was dissolved in 95% ethanol solution at a concentration of 5 mg/mL and stored in brown bottles to be used as a stock solution. Then, 0.2, 0.4, 1, 2, and 3.33 mL of curcumin stock solution was poured into 10 mL of Car/SPI solution (SPI 5 mg/mL; Car 5 mg/mL) and homogenized for 10 min under pH 3.5 using a high-speed shear homogenizer (10,000 rpm). The residual ethanol in the system was removed with a rotary evaporator to obtain a final aqueous dispersion of Car/SPI/C composite nanoparticles. The mass ratios of SPI to curcumin were 50:1, 25:1, 10:1, 5:1, and 3:1.

#### 3.2.4. Curcumin-Loaded Fuc/Car-Stabilized SPI Nanoparticles (Fuc/Car/SPI/C) under Different Car/SPI Ratios

We poured 1 mL of curcumin stock solution (5 mg/mL) into a beaker containing 10 mL of SPI/Car solution (SPI 5 mg/mL; Car 5 mg/mL) under pH 3.5 and was homogenized for 10 min using a high-speed shear homogenizer (10,000 rpm). Next, 10 mL of Fuc solution (concentrations of 0, 0.5, 1, 1.5, 2, and 2.5 mg/mL), which was used for stabilizing, was poured into 10 mL of Car/SPI nanoparticle dispersion and homogenized (10,000 rpm) for 5 min. The residual ethanol in the system was removed with a rotary evaporator to obtain a final aqueous dispersion of Fuc/Car/SPI/C composite nanoparticles, where the mass ratios of SPI to Fuc were 1:0, 1:0.1, 1:0.2, 1:0.3, 1:0.4, and 1:0.5.

### 3.3. Determination of Particle Size, Zeta Potential, and PDI

The particle size, zeta potential, and PDI of composite nanoparticle were determined using a Malvern Delsa Nano system (Malvern Instruments Ltd., Marvin, UK) according to the method from Liu et al. [26]. For comparison, the SPI/C nanoparticles were also investigated. Specifically, 1 mL of curcumin stock solution (5 mg/mL) was poured into 10 mL of SPI solution (5 mg/mL) under pH 3.5 and was homogenized for 10 min.

The instrument was set to 25 °C and preheated for 30 min. The samples were poured into the cuvette and stirred at 200 rpm for 5 min before we began the measurement. The shading ratio was set at 10–20%. The average particle size, zeta potential, and PDI were calculated and obtained using light scattering data processing software (Zetasizer, version 7.11).

All the experiments were carried out in triplicate and 25 runs were performed for each solution.

### 3.4. Determination of Turbidity

The turbidity of the prepared colloidal dispersion was measured using the colorimetric method [33]; that is, the absorbance of different nanoparticles was tested at 460 nm using a UV-Vis spectrophotometer (723PC, Hengping Scientific Instrument Co., Ltd., Shanghai, China), which was used to indicate the turbidity.

### 3.5. Calculation of the Encapsulation Efficiency and Loading Capacity

Determination of free curcumin [9]: 1 mL of sample was added to 3 mL of dichloromethane and extracted by shaking for 2 min. The solution was left to stand to facilitate layering. The lower layer was taken and diluted with anhydrous ethanol. The absorbance at 427 nm was measured with a UV-Vis spectrophotometer (723PC, Hengping Scientific Instrument Co., Ltd., Shanghai, China) to calculate the curcumin content according to the standard curve: Y = 0.1575X − 0.0155 (R^2^ = 0.9998) (detailed in Figure S1). Here, Y is the absorbance value, and X is the content of the curcumin (μg/mL).

Determination of total curcumin [9]: 1 mL of the sample was added to 3 mL of anhydrous ethanol and mixed thoroughly under sonication before centrifuging (22331 Hamburg, Eppendorf Ltd., Hamburg, Germany) at 8000 rpm for 3 min. The supernatant was transferred to determine the curcumin content. The encapsulation efficiency and loading capacity of curcumin were calculated as follows:

(1) $$Encapsulation\;efficiency\left(\%\right)=\left(1-\frac{{curcumin}_{free}}{{curcumin}_{total}}\right)\times 100$$

(2) $$Loading\;capacity(\%)=\frac{encapsulated\;curcumin}{the\;mass\;of\;nanoparticles}\times 100$$

### 3.6. Scanning Electron Microscopy (SEM)

The nanoparticles were observed using SEM (Zeiss Sigma 300, Zeiss Ltd., Oberkochen, Germany). The solutions of samples were centrifuged at 10,000 rpm for 5 min to isolate the precipitate. Thereafter, the supernatant was freeze-dried to obtain nanoparticle powder samples. These samples were gently spread on the conductive gel, and the morphology of samples was observed in high vacuum mode at 5.0 kV with a magnification of 50,000 after conductive coating.

### 3.7. Fluorescence Spectroscopy and FTIR

The binding mechanism between curcumin and composite nanoparticles was investigated via fluorescence spectroscopy in accordance with the method of Tiwari et al. [34] with some modifications. Briefly, three nanoparticles, namely, SPI/C, Car/SPI/C, and Fuc/Car/SPI/C, were used to determine the steady-state fluorescence spectrum of curcumin in the 460–630 nm range of emission wavelengths using a Shimadzu RF-5301 fluorescence spectrophotometer (Shimadzu Co., Kyoto, Japan). The excitation wavelength was set at 427 nm, whereas the emission slit and excitation slit widths were both 2.5 nm. The curcumin in aqueous solutions was used as a control group for comparison.

The interactions between proteins and polysaccharides in the nanoparticles were determined via Fourier transform infrared spectroscopy (FTIR) using a Nicolet iS10 (Nicolet iS10, Madison, WI, USA) spectrometer. The dried powder samples were individually ground with spectroscopic-grade KBr at a ratio of 1:200 and pressed into pellets. The physical mixture of the four components (C, SPI, Car and Fuc), present in the nanoparticles in equivalent ratios, was determined as a control. The wavelength range measured was 4000 to 600 cm^−1^, with a scanning frequency of 32 and a resolution of 4 cm^−1^.

### 3.8. Photochemical Stability

According to Zheng et al.’s method [35] with slight modifications, the photochemical stability of SPI/C, Car/SPI/C, and Fuc/Car/SPI/C between pure curcumin (as a control group) was compared to evaluate the influence of nanoencapsulation on the stability of curcumin. The powder samples were exposed to strong light (4400 lux) and soft light (2200 lux) at room temperature (25 ± 2 °C). Samples under strong light conditions were taken per 8 and 16 h under soft light condition. The curcumin content was determined as described in Section 3.5, and the retention rates were calculated for comparison. The formula for curcumin retention was as follows:

(3) $$Retention\;rate\;of\;curcumin\left(\%\right)=\frac{{curcumin}_{after\;a\;period\;of\;treatment}}{{curcumin}_{original}}\times 100$$

### 3.9. Thermal Stability

The effects of three different heat treatment conditions on the stability of curcumin in lyophilized samples were investigated in accordance with Li et al.’s method [36] with some modifications. The curcumin retention within samples heated at 80 °C, 60 °C, and 40 °C using an air-dry oven (DHG-903385-III, Jing Hong Laboratory Equipment Co., Ltd., Shanghai, China) was measured every 2, 1, and 1 day, respectively. Pure curcumin was used as a control group to compare the difference in the amount of curcumin retained between samples.

### 3.10. Controlled Release of Curcumin under Simulated Gastrointestinal Fluid

The kinetic release profile of curcumin from composite nanoparticles was evaluated using the dialysis membrane method under simulated gastrointestinal according to a previous study [37]. Briefly, the simulated gastric fluid (SGF, pH 4 with 1 mg/mL pepsin) and simulated gastric fluid (SIF, pH 7.4 with 4 mg/mL pancreatin) were first mixed with an equal volume of ethanol to prepare the release medium with sink condition (50% ethanol) for curcumin. The dialysis bag with 10,000 Da molecular cutoff containing the 4 mL of freshly prepared each sample (curcumin-loaded nanoparticles or free curcumin) was first incubated in 80 mL SGF for 2 h (37 °C, 100 rpm) and then transferred to 80 mL SIF for 4 h incubating. At last, 1 mL of release medium was collected at per half hour for curcumin measurement and fresh release medium was replenished to maintain the constant volume. All the experiments were carried out in triplicate.

### 3.11. Antioxidant Properties of Curcumin-Loaded Nanoparticles

#### 3.11.1. DPPH Radical Scavenging Activity

The DPPH radical scavenging capacity of the different samples was determined using a modified method based on a previous report [38]. Encapsulated curcumin and pure curcumin were prepared at the same concentration and mixed with 1.5 mL of ethanolic DPPH solution (0.2 mM); they then reacted in the dark for 30 min at room temperature. Subsequently, the absorbance was measured at 517 nm. The DPPH radical scavenging activity was then calculated using the Formula (4):

(4) $$Radical\;scavenging\;activity\left(\%\right)=\frac{A_{C}-As}{A_{C}}\times 100$$

where A_C_ and A_S_ are the absorbance of control and sample solutions, respectively. Scavenging activity (SC_50_) means the concentration of the sample required to reduce scavenging by 50%, which is used to compare the level of free radical scavenging activity of different samples.

#### 3.11.2. Reducing Power Capacity

The reducing power of various samples was evaluated using a modified version of the method developed by Huang et al. [38]. To begin, 1 mL of each sample with a consistent curcumin concentration of 200 μg/mL was combined with 2.5 mL of a phosphate-buffered solution (PBS, 0.2 M, pH 6.6) and 2.5 mL of a potassium ferricyanide solution (1%, *w*/*v*). The mixture was then incubated at 50 °C for 20 min. To halt the reaction, 2.5 mL of a trichloroacetic acid (TCA, 10%) solution was added, and the system was subjected to centrifugation at 5000 rpm for 5 min. Subsequently, the supernatant (2.5 mL) was taken out and mixed with 2.5 mL of distilled water and 0.5 mL of ferric chloride solution. After a further 10 min of incubation at room temperature, the absorbance was measured at 700 nm using a UV–visible spectrophotometer (723PC, Hengping Scientific Instrument Co., Ltd., Shanghai, China).

## 4. Conclusions

In summary, a curcumin-loaded SCI nanoparticle with improved dispersibility and stability was successfully fabricated via Fuc/Car co-stabilized coating. The results show that the mass ratio of SPI to each component (Car, Cur, Fuc) and the aggregated pH had a profound influence on the physicochemical properties of the different nanoparticles (SPI/C, Car/SPI/C, Fuc/Car/SPI/C). Compared with SPI/C, the addition of Car or Fuc/Car dramatically improved the redispersibility of the freeze-dried nanoparticles, particularly the latter, as indicated by the high zeta potential of −29.67 mV and low PDI of 0.12 at the pH of 3.5. The 3D network of Fuc/Car can offer a continuous membrane to entirely encapsulate the SPI/Cs with a 95.28% encapsulation efficiency for curcumin and 4.16% loading capacity. The self-assembled mechanism was also validated by fluorescence spectroscopy, which indicated that Fuc/Car can provide a more hydrophobic conjugation environment for SPI/C. In particular, Fuc/Car plays an important role in dramatically enhancing the stability and antioxidant activity of curcumin, and providing a sustained release in simulated gastric and intestinal fluids. Overall, our SPI nanoparticle with a 3D coating network (Fuc/Car) formed by simply blending not only widens the scope of Fuc applications but also provides a promising and effective nanocarrier for the encapsulation of curcumin used in water-soluble food matrices and drugs.

## Figures and Tables

**Table 1:** Effects of the mass ratio of SPI to Car on nanoparticle size, zeta potential, and the turbidity of the solution *.

SPI:Car (*w*/*w*)	Particle Size (nm)	Zeta Potential (mV)	Turbidity
1:0	1069.49 ± 1.57 ^a^	17.58 ± 0.81 ^a^	1.75 ± 0.01 ^e^
1:0.25	344.04 ± 9.26 ^c^	−18.17 ± 0.84 ^d^	1.85 ± 0.04 ^d^
1:0.5	343.14 ± 7.63 ^c^	−22.23 ± 1.02 ^bc^	2.43 ± 0.05 ^b^
1:1	329.82 ± 6.58 ^e^	−24.12 ± 1.14 ^b^	2.58 ± 0.01 ^a^
1:1.25	336.97 ± 8.47 ^d^	−21.76 ± 0.98 ^bc^	2.34 ± 0.04 ^c^
1:1.5	355.03 ± 9.83 ^b^	−21.24 ± 0.74 ^c^	2.31 ± 0.04 ^c^

* Different letters represent significant differences between groups (*p* < 0.05).

**Table 2:** Effects of the pH value on nanoparticle size, zeta potential, and turbidity *.

pH	Particle Size (nm)	Zeta Potential (mV)	Turbidity
4.5	356.94 ± 1.07 ^a^	−15.24 ± 0.82 ^d^	2.45 ± 0.02 ^b^
4.0	334.76 ± 2.80 ^c^	−21.71 ± 0.88 ^b^	3.14 ± 0.03 ^a^
3.5	320.15 ± 1.53 ^e^	−24.78 ± 1.21 ^a^	3.21 ± 0.02 ^a^
3.0	324.42 ± 1.09 ^d^	−23.56 ± 1.10 ^ab^	3.12 ± 0.02 ^a^
2.5	344.97 ± 3.10 ^b^	−18.42 ± 0.82 ^c^	2.46 ± 0.01 ^b^

* Different letters represent significant differences between groups (*p* < 0.05).

**Table 3:** Effects of the mass ratio between SPI and curcumin on the physicochemical properties of curcumin-loaded Car-SPI nanoparticles *.

SPI:Curcumin (*w*/*w*)	Particle Size (nm)	Zeta Potential (mV)	Turbidity	Encapsulation Efficiency (%)
50:1	420.41 ± 0.04 ^a^	−23.71 ± 1.23 ^ab^	4.17 ± 0.03 ^c^	87.55 ± 0.20 ^a^
25:1	419.23 ± 0.85 ^ab^	−24.26 ± 1.17 ^a^	4.19 ± 0.05 ^bc^	86.60 ± 0.31 ^b^
10:1	419.11 ± 0.33 ^ab^	−25.07 ± 1.31 ^a^	4.23 ± 0.02 ^bc^	86.51 ± 0.17 ^b^
5:1	417.88 ± 0.52 ^b^	−22.72 ± 1.02 ^ab^	4.28 ± 0.06 ^b^	85.58 ± 0.23 ^c^
3:1	418.10 ± 0.61 ^b^	−21.56 ± 0.98 ^b^	5.04 ± 0.08 ^a^	78.88 ± 0.30 ^d^

* Different letters represent significant differences between groups (*p* < 0.05).

**Table 4:** Effects of the mass ratio between SPI and Fuc on the physicochemical proper of curcumin-loaded Fuc-Car-SPI microcapsules *.

SPI:Fuc (*w*/*w*)	Zeta Potential (mV)	Turbidity	Loading Capacity (%)
1:0	−25.07 ± 1.31 ^c^	4.23 ± 0.02 ^d^	5.01 ± 0.09 ^a^
1:0.1	−27.24 ± 1.01 ^abc^	6.05 ± 0.10 ^c^	4.73 ± 0.09 ^b^
1:0.2	−27.83 ± 1.28 ^ab^	6.16 ± 0.01 ^bc^	4.56 ± 0.06 ^c^
1:0.3	−28.31 ± 1.43 ^ab^	6.23 ± 0.01 ^b^	4.34 ± 0.05 ^d^
1:0.4	−29.67 ± 1.38 ^a^	6.30 ± 0.01 ^ab^	4.16 ± 0.04 ^e^
1:0.5	−26.02 ± 1.15 ^bc^	6.43 ± 0.01 ^a^	4.12 ± 0.07 ^e^

* Different letters represent significant differences between groups (*p* < 0.05).

## Data Availability

Data are contained within the article and Supplementary Material.

## Supplementary Materials

The following supporting information can be downloaded at: https://www.mdpi.com/article/10.3390/ph17040534/s1, Figure S1: The standard curve of curcumin; Figure S2: Optical microscope images of Car/SPI nanoparticles under different pHs. (A) pH = 4.5; (B) pH = 4; (C) pH = 3.5; (D) pH = 3.0; (E) pH = 2.5; Figure S3: The average particle size and encapsulation efficiency of nanoparticles. * indicates a significant difference between groups of 1:0.4 and 1:0 in particle size, * *p* < 0.05; ^△^ represents a significant difference between groups of encapsulation efficiency, ^△^ *p* < 0.05, ^△△^ *p* < 0.01, ^△△△^ *p* < 0.001.
