# Supplementary material for: An Effective Method to Prepare Curcumin-Loaded Soy Protein Isolate Nanoparticles Co-Stabilized by Carrageenan and Fucoidan
Source: Pharmaceuticals (Basel). 2024 Apr 21;17(4):534. doi: 10.3390/ph17040534 (PMC11055026; doi:10.3390/ph17040534)

Supplementary Material

Figure captions

Figure S1-The standard curve of curcumin

Figure S2-Optical microscope images of Car/SPI nanoparticles under different pHs. (A) pH=4.5; (B) pH=4; (C) pH=3.5; (D) pH=3.0; (E) pH=2.5

Figure S3-The average particle size and encapsulation efficiency of nanoparticles. * indicates a significant difference between groups of 1:0.4 and 1:0 in particle size, *$P<0.05$; △ represents a significant difference between groups of encapsulation efficiency, $^{\triangle}P<0.05$, $^{\triangle\triangle}P<0.01$, $^{\triangle\triangle\triangle}P<0.001$.

Figure S1

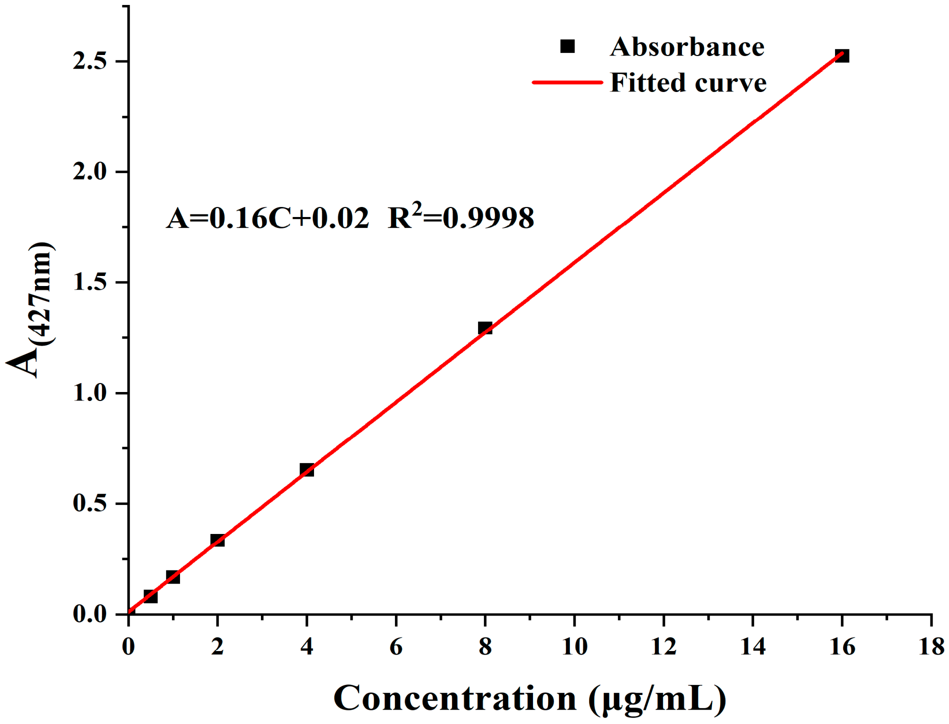

Figure S2

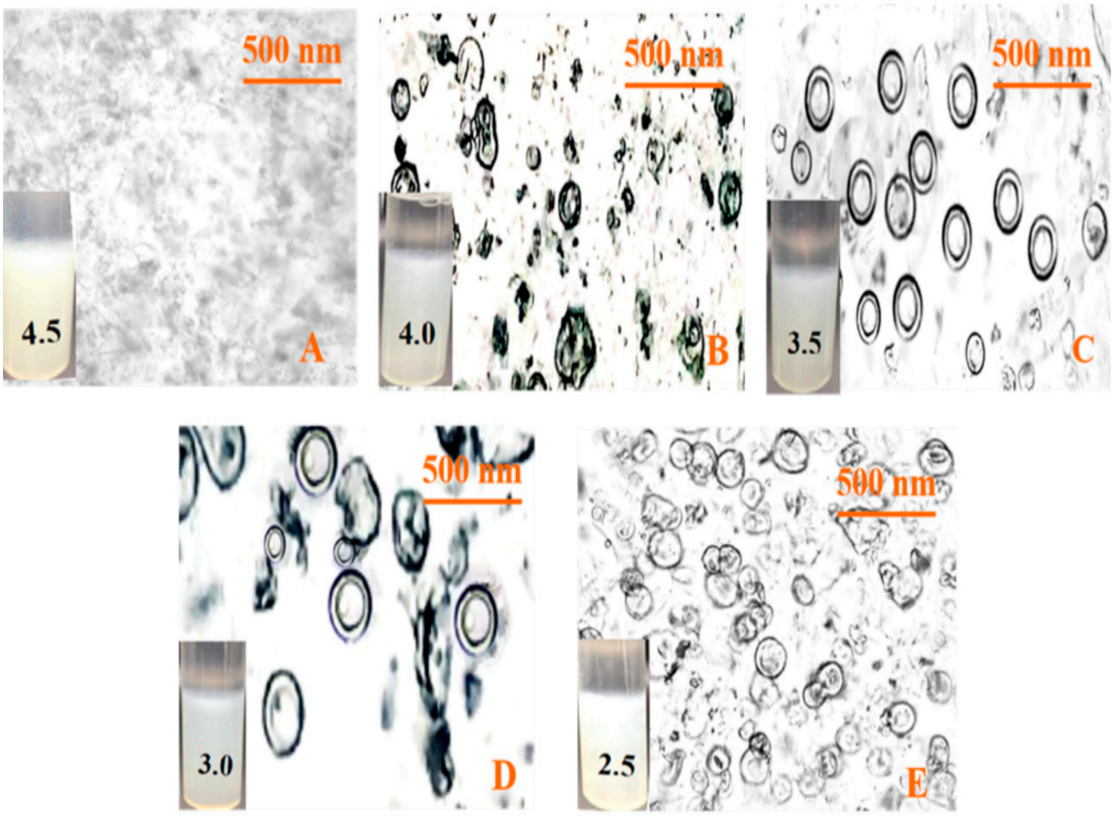

Figure S3

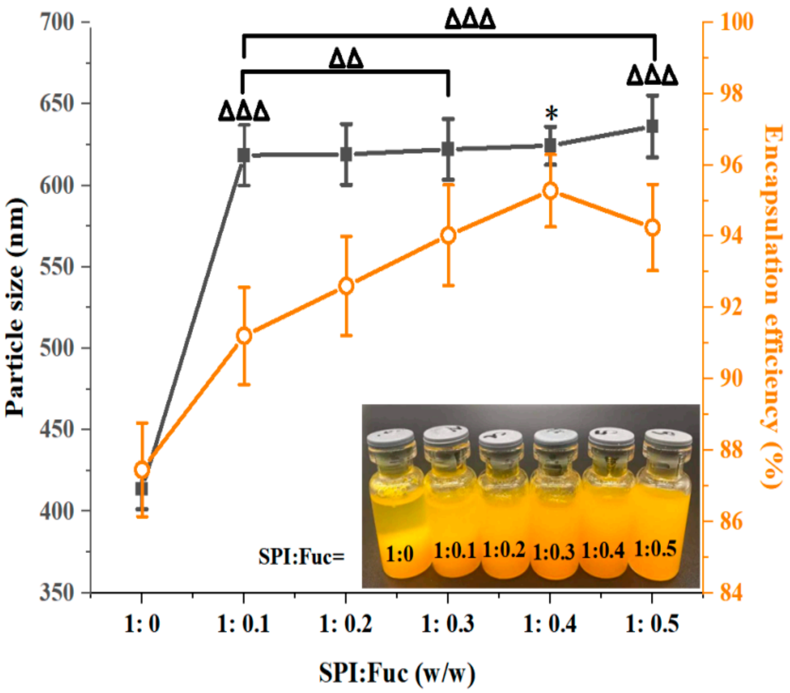

Supplement: Supplementary file 1 [file pharmaceuticals-17-00534-s001.zip › pharmaceuticals-2958455-supplementary.pdf]
